# Anterior Paraventricular Thalamus to Nucleus Accumbens Projection Is Involved in Feeding Behavior in a Novel Environment

**DOI:** 10.3389/fnmol.2018.00202

**Published:** 2018-06-07

**Authors:** Jingjing Cheng, Jincheng Wang, Xiaolin Ma, Rahim Ullah, Yi Shen, Yu-Dong Zhou

**Affiliations:** ^1^Department of Neurobiology, Key Laboratory of Medical Neurobiology of Ministry of Health, Zhejiang Province Key Laboratory of Neurobiology, Zhejiang University School of Medicine, Hangzhou, China; ^2^Department of Endocrinology, Children’s Hospital, Zhejiang University School of Medicine, Hangzhou, China

**Keywords:** novelty-suppressed feeding, novelty seeking, anxiety, NAc, aPVT

## Abstract

Foraging food in a novel environment is essential for survival. Animals coordinate the complex motivated states and decide whether to initiate feeding or escape from unfamiliar scenes. Neurons in the paraventricular thalamic nucleus (PVT) receive multiple inputs from the hypothalamus, forebrain, and caudal brainstem that are known to regulate feeding behavior. The PVT neurons also project to the forebrain regions that are involved in reward and motivation. Notably, the PVT neurons projecting to the nucleus accumbens (NAc) are activated when an incentive stimulus is presented. Optogenetic activation of the PVT-NAc path has been shown to increase the motivation for sucrose-seeking in instrumental tasks. However, how the PVT circuitry regulates the feeding behavior in a novel environment remains largely obscure. In the present study, we found that the activity of glutamatergic neurons in the anterior PVT (aPVT) projecting to the NAc dictates the novelty-suppressed feeding behavior in mice. Optogenetic activation of the aPVT-NAc projection increased the feeding time and food consumption in mice under a moderate food restriction in a novel open field where the food was placed in the central area. The exploratory and anxiety-like behaviors, however, were not altered by the aPVT-NAc activation. Our work reveals that activation of the aPVT-NAc pathway in mice generates a motivation to consume food in a novel environment.

## Introduction

The initiation of feeding behavior in a natural environment is usually dependent on food reward value. While hunger provides the primary motivation to eat, food-seeking (wanting/liking) and ingestive behaviors may also be triggered by an anticipation of high food reward value in a particular environmental context (Harb et al., 2014). In a free-living situation, the risk of foraging food in an unsafe environment and the associated food reward value must be evaluated beforehand (Lockie et al., 2017). Such a decision-making process recruits a complicated, organized neuronal circuit that harbors feeding and reward centers. Impairment in this decision-making circuitry may contribute to various eating disorders such as anorexia and uncontrollable overeating.

Increasing evidence suggests that the paraventricular thalamus (PVT) is an important brain region responsible for the decision-making process. As a stable midline structure throughout mammalian evolution, the PVT plays a pivotal role in integrating information that is related to appetite, motivation, and aversion (Millan et al., 2017). Like other thalamic nuclei, the PVT consists primarily of glutamatergic neurons, with neuronal subpopulations classified according to calcium binding protein expression patterns or diverse neuropeptide immunoactivities (Kirouac, 2015). Direct stimulation of the PVT glutamatergic neurons reduces food intake (Zhang and van den Pol, 2017), whereas activation of GABA_A_ receptors in the PVT dose-dependently increases food intake (Stratford and Wirtshafter, 2013). The PVT is also considered to balance the defensive action and reward approaching depending on the demand of the task (Choi and McNally, 2017). The connection between the PVT and amygdala is linked to fear learning and expression and fear retrieval (Penzo et al., 2015). Based on the findings that (1) the PVT-hypothalamus reciprocal projections are related to feeding behavior (Stratford and Wirtshafter, 2013) and (2) the PVT-prefrontal cortex circuit is responsible for decision-making (Pinto et al., 2003; Li and Kirouac, 2008; Dong et al., 2017), we propose that the PVT may represent a crucial element in the neuronal circuitry regulating the motivated feeding behaviors. Moreover, the PVT innervates the nucleus accumbens shell (NAcSh) via a dense bundle of glutamatergic fibers (Parsons et al., 2006; Vertes and Hoover, 2008). Activation of the PVT GLUT2 neurons projecting to the NAc increases sucrose- but not saccharin-seeking behaviors in instrumental tasks (Labouebe et al., 2016), indicating that the PVT-NAc circuit is responsible for the positive motivation to seek sucrose. In another study, photoinhibition of the PVT-NAc pathway increases sucrose seeking only when reward is omitted in a conditional operant task (Do-Monte et al., 2017). These pieces of evidence suggest the PVT-NAc circuit is responsible for motivated behaviors. However, the role of the PVT-NAc circuit in the feeding behavior under approach-avoidance conflict remains unknown.

Here, by using a modified open field test (Lockie et al., 2017), we demonstrated an increase in the c-Fos expression in the NAc and anterior PVT (aPVT) when a mouse was performing the novelty-suppressed feeding (NSF) task. We further established that the NAc medium spiny neurons (MSNs) were innervated by glutamatergic terminals from neurons in the aPVT by using a combination of the anterograde and retrograde virus tracing and electrophysiological recording methods. Photo-stimulation of the aPVT-NAc circuit in mice significantly increased the time spent in the center area and food consumption in the NSF test. Activation of the aPVT-NAc circuit, however, did not change the novelty seeking (NS) behavior, locomotor activity, and anxiety level in mice. Together, our findings indicate that the aPVT-NAc circuit is involved in the motivated feeding behavior in an open field.

## Materials and Methods

### Animals

All procedures were carried out in accordance with the National Institutes of Health Guidelines for the Care and Use of Laboratory Animals and were approved by the Animal Advisory Committee at Zhejiang University. C57BL/6J male mice (3–4 months, 26–30 g) were group housed in a temperature-controlled room on a 12 h/12 h light/dark cycle. Food and water were available *ad libitum*. Mice used for behavioral tests were handled twice a day, for 3 consecutive days before the behavioral tests.

### Surgery

Mice were anesthetized with isoflurane (3% for induction, 1.5-2.0% thereafter) and fixed in a stereotaxic apparatus (RWD Life Science, China). The position of the head was horizontally adjusted. A stereo microscope (RWD Life Science, China) was used to observe the small incision exposed above the skull. All mice were allowed to recover from the surgery for at least 7 days. For virus injections, a microsyringe pump (kdScientific,United States) was used to control injection speed at a rate of 0.1 μl/min through a 10 μl micro syringe (#701, Hamilton, United States) loaded with a beveled 32-gauge microinjection needle. Mice were injected AAV2/9-CaMKIIα-ChR2-mCherry or AAV2/9-CaMKIIα-mCherry virus (0.6 μl, titer: 1×10^12^ vg/ml; BrainVTA, China) in the aPVT at a stereotaxic coordinate of anteroposterior (AP) -0.4 mm, mediolateral (ML) -0.6 mm, and dorsoventral (DV) -3.6 mm based on the mouse brain atlas of Paxinos and Franklin (2008). A 10° needle angle was adopted to avoid damage to the superior sagittal sinus. The retro-AAV2-CMV-GFP virus (0.2 μl, titer: 1×10^13^ vg/ml; Taitool Bioscience Co. Ltd., China) was unilaterally injected into the NAc at a stereotaxic coordinate of AP +1.5 mm, and ML +0.8 mm or -0.8 mm, and DV -4.2 mm. Infections with AAVs were allowed for at least 4 weeks for maximal viral expressions in neurons in the target areas. To implant a fiber cannula above the NAc, a fiber optic cannula with Ø1.25 mm ceramic ferrule was attached to a probe holder and unilaterally inserted into a location (+1.5 mm AP, +0.8 mm or -0.8 mm ML, and -4.0 mm DV). The cannula was then anchored to the skull with metal screws and secured by dental cement. A multimode fiber (Ø200 μm, NA 0.22) patch cable was attached to the cannula when an optogenetic experiment was conducted. Each mouse was assured for the virus expression and fiber placement in the proper areas after the behavioral tests, and the data from unqualified mice were excluded.

### Open Field Test

The open field apparatus consists of an open Plexiglas box (45 × 45 × 45 cm^3^) with 250 lux of illuminance at the center. Mice were placed in a corner of the open field apparatus and allowed to explore for 5 min. The time spent in the center area, the number of entries to the center area, and the total locomotor distance were recorded. The apparatus was cleaned with 75% ethanol between tests.

### NS and NSF Tests

The test apparatus is the same as used in the open field test. A novel object (a cylinder, 5 cm in diameter and 10 cm high; the NS test) or a pre-weighed wet food pellet (the NSF test) was placed in the center of the apparatus. After an open field test session, mice were then returned to the open field for an additional 5 min NS or NSF test. In the NS test, the seeking time in center, the center entries, and the total locomotor distance were recorded by the ANY-maze system (Stoelting, United States). Mice were fasted for 16–18 h before the NSF test. In the NSF test, the food consumption, the feeding time in center, and the center entries were recorded. Rearing was manually counted for exploratory behavior comparison. 473 nm laser pulses (4–6 mW, 20 Hz, 5 ms pulse width) were switched on for 5 min to activate the aPVT-NAc projection.

### Normal Feeding Test

Mice were fasted for 16–18 h before the normal feeding test. Mice were first allowed to habituate in a chamber (30 × 12 × 15 cm^3^) with two empty plastic dishes (diameter 2.5 cm) placed in opposite corners for a period of 10 min. One wet food pellet was alternatively placed in the dishes between trials, and mice were then allowed to freely approach the pellet for 5 min with or without activating the aPVT-NAc projection by 473 nm laser pulses (4–6 mW, 20 Hz, 5 ms duration). The food consumption was recorded and the time spent eating was analyzed manually during the 5-min period. The apparatus was cleaned with 75% ethanol between tests.

### Elevated Plus-Maze (EPM) Test

The maze consists of two open arms (30 × 5 cm^2^) and two closed arms with the same size enclosed by high black walls (30 × 5 × 15 cm^3^) in an arrangement that the two arms of each type are opposite to each other. The maze is elevated 65 cm above the ground and a video camera is fixed above the maze to record the movements for analysis. The behavioral experiments were conducted in a quiet room illuminated by a dim light. Mice were adapted in the open field for 5 min to reduce the anxiety. Each animal was placed in the same position and allowed to explore for 5 min. Time in the open arms and number of entries into the open arms were analyzed using the video-based ANY-maze system (Stoelting, United States). Mice fell off the maze during the test were excluded. The maze was cleaned with 75% ethanol between tests to prevent olfactory cues from influencing the behavior of subsequently tested animals.

### Light–Dark Box Test

The light–dark box test was performed as previously described (Crawley and Goodwin, 1980) with a few modifications. A Plexiglas box (50 × 25 × 25 cm^3^) containing a dark and an illuminated compartment of the same size (25 × 25 × 25 cm^3^) with a 5 × 5 cm^2^ opening in between was used in the test. After an adaptation period of more than 1 h in the test room, mice were placed in the dark compartment and allowed to freely explore the box for 10 min with or without photostimulating the aPVT-NAc pathway. The number of fecal boli produced, the latency of entering into the light compartment, and the time spent in the light compartment were recorded. The apparatus was cleaned with 75% ethanol between tests.

### Immunohistochemistry

Immunohistochemistry was performed as previously described (Li et al., 2016). Briefly, 90 min after the open field (OFT group), novelty-seeking (NST group), or novelty-suppressed feeding (NSF group) test, when c-Fos expression in activated neurons reaches to the peak, mice were deeply anesthetized and perfused transcardially with PBS, followed by 4% paraformaldehyde (PFA) in PBS. Brains were extracted and post-fixed in 4% PFA overnight and then moved to 30% sucrose in PBS. After brains were saturated, coronal brain sections (30 μm thickness) were cut using a freezing microtome (CM30503, Leica Microsystems, Germany). Sections were processed as free-floating sections. After being blocked in PBS containing 10% normal donkey serum (NDS; 017-000-121, Jackson ImmunoResearch, United States), 1% bovine serum albumen (BSA; A2153, Sigma, United States), 0.3% Triton X-100 in PBS for 1 h at room temperature (RT), tissue sections were incubated with an anti-c-Fos antibody (1:800; #2250, Cell Signaling Technology, United States) for 72 h at 4°C. Sections were then washed with PBS (15 min, three times) and incubated with donkey anti-rabbit Alexa Fluor 488 (1:1000; A-21206, Thermo Fisher Scientific, United States) at RT for 1 h. After washing with PBS three times, sections were mounted onto glass slides with 60% glycerol in PBS. All Images were captured using a Nikon A1 laser-scanning confocal microscope (Nikon, Japan) through a 20X objective (numerical aperture 0.75) under a fixed set of settings. The number of immune-positive cells was counted and analyzed using ImageJ (NIH, United States).

### Electrophysiology

4 weeks after injection of the AAV-CaMKIIα-ChR2-GFP virus in the aPVT, mice were anesthetized with isoflurane and brains were quickly removed and transferred to a chamber filled with ice-cold cutting solution (in mM: 234 Sucrose, 5 KCl, 1.25 NaH_2_PO_4_, 5 MgSO_4_, 26 NaHCO_3_, 25 Dextrose, 1 CaCl_2_, bubbled with 95% O_2_/5% CO_2_). Coronal slices (300 μm) were cut with a vibratome (VT 1200S, Leica, Germany) and then incubated in oxygenated (95% O_2_/5% CO_2_) artificial cerebrospinal fluid (ACSF, in mM: 124 NaCl, 2 KCl, 1.25 KH_2_PO_4_, 2 MgSO_4_, 2 CaCl_2_, 26 NaHCO_3_, and 10 D-(+)-Glucose, pH 7.4). Slices were allowed to recover for ∼30 min in ACSF at 32°C, and subsequently for 1 h at RT. Brain slices were then transferred to a recording chamber and perfused continuously with ACSF at 35–36°C bubbled with 95% O_2_/5% CO_2_ to ensure adequate oxygenation. ChR2-expressing area in the NAc was first identified under the epifluorescence configuration and then visualized under the infrared differential interference contrast optics via an upright microscope (BX51WI, Olympus, Japan). Whole-cell patch clamp recordings were obtained from the NAc MSNs adjacent to ChR2-labeled fibers based on their location and morphology. Borosilicate glass (Sutter instruments, United States) patch electrodes (3–5 MΩ) were pulled with a horizontal pipette puller (P97, Sutter instruments, United States) and filled with an artificial intracellular fluid (in mM: 120 K-gluconate, 15 KCl, 10 HEPES, 4 Mg-ATP, 0.3 Tris-GTP, 0.5 EGTA, adjusted to pH 7.3 with KOH, 285–290 mOsm). Pipettes were connected to the headstage of a Heka EPC 10 amplifier (Heka Elektronik, United States), and fast and slow capacitance as well as series resistance compensations were carefully adjusted. Current was injected into cells to maintain the baseline membrane potential at ∼-70 mV in the whole-cell current-clamp mode using the potassium gluconate-based pipette solution mentioned above. To record photo-evoked synaptic events, terminals expressing ChR2 were activated by 1 Hz 470-nm laser trains with a pulse width of 5 ms. The GABAergic blocker bicuculline methiodide (BMI, 10 μM) and the AMPA receptor blocker 6,7-dinitroquinoxaline-2,3-dione (DNQX, 10 μM) were bath applied. Electrophysiological data were analyzed using IGOR Pro 6 (WaveMetrics, United States) as described before (Shen et al., 2016).

### Statistical Analysis

GraphPad Prism^TM^ 5.0 (version 5.0; GraphPad Software Inc., United States) and SigmaStat (Systat Software Inc., United States) were used for data display and statistical analysis. One-way ANOVA followed by Bonferroni correction was used in the analysis of c-Fos staining. Two-way ANOVA followed by *post hoc* Holm–Sidak multiple comparison test was used in the analysis of all behavioral results. Significance was reported as *p* < 0.05, and data were expressed as mean ± SEM.

## Results

### Enhanced aPVT and NAc Neuronal Activities Are Associated With NSF Behavior

**FIGURE 1:**
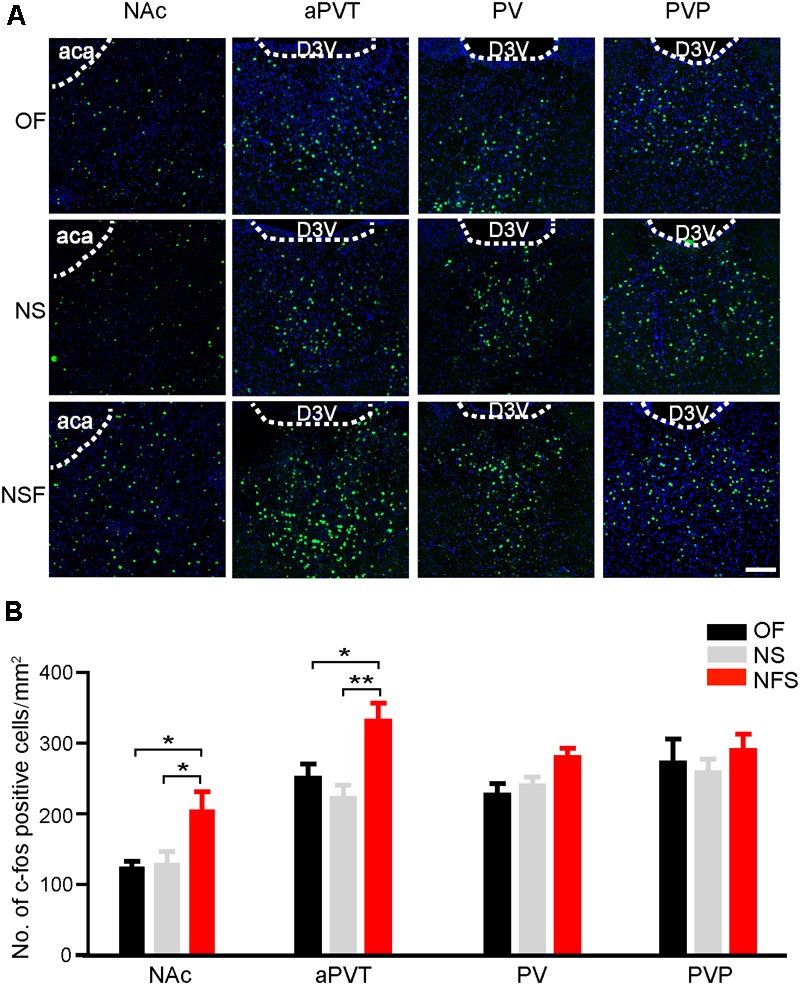
Enhanced aPVT and NAc neuronal activities are associated with NSF behavior. **(A)** Representative images of c-Fos immunostaining in the NAc, aPVT, PV, and PVP in mice from the OF, NS, and NSF groups. Scale bar = 100 μm. **(B)** Quantification of c-Fos positive cell numbers in the NAc (*n* = 13, 22, and 16 for OF, NS, and NSF groups, respectively), aPVT (*n* = 8, 8, and 9 for OF, NS, and NSF groups, respectively), PV (*n* = 5, 7, and 8 for OF, NS, and NSF groups), and PVP (*n* = 5, 8, and 8 for OF, NS, and NSF groups, respectively) in mice from the OF, NS, and NSF groups. ^∗^*p* < 0.05, ^∗∗^*p* < 0.01 (one-way ANOVA followed by Bonferroni correction). D3V, dorsal 3rd ventricle; aca, anterior part of anterior commissure. Data are mean ± SEM.

We first aimed to determine if the neuronal activities in the PVT and NAc were altered in mice that had performed the NSF task. Mice were handled for 3 consecutive days and moderately food restricted for 16–18 h before the behavioral tests to decrease the stress-related neuronal activation and to facilitate food-seeking. A pellet of food or a novel object was placed in the center area of the open field apparatus. Mice were then allowed to freely explore in the open field for 5 min. Mice approaching the food pellet (NSF group) or the object (NS group) were sacrificed 90 min later for immunostaining with an antibody against a neuronal activity maker c-Fos. Naïve mice explored freely in the open field (OF group) were used as baseline controls for c-Fos expression. Mice frozen in the corner were excluded. As shown in **Figure 1**, mice in the NSF group exhibited an increase in the number of c-Fos positive neurons in the NAc in comparison to the mice in the NS or OF group (one-way ANOVA with Bonferroni correction; *F*_(2,48)_ = 4.506, *p* < 0.05, NSF vs. OF group; *p* < 0.05, NSF vs. NS group; *n* = 13, 22, and 16 for OF, NS, and NSF groups, respectively; **Figure 1**). Since the PVT is a complicated nucleus that is comprised of functionally distinct anterior (aPVT), middle (PV), and posterior (PVT) subregions, we further examined the c-Fos expression patterns within the PVT. Interestingly, we found a notable increase in the number of c-Fos positive neurons in the aPVT (one-way ANOVA with Bonferroni correction; *F*_(2,22)_ = 6.897, *p* < 0.05, NSF vs. OF group; *p* < 0.01, NSF vs. NS group; *n* = 8, 8, and 9 for OF, NS, and NSF groups, respectively), whereas no changes in c-Fos positive neuron numbers were found in the PV (one-way ANOVA with Bonferroni correction; *F*_(2,17)_ = 4.057, *p* > 0.05; *n* = 5, 7, and 8 for OF, NS, and NSF groups, respectively) and PVP (one-way ANOVA with Bonferroni correction; *F*_(2,18)_ = 0.491, *p* > 0.05; *n* = 5, 8, and 8 for OF, NS, and NSF groups, respectively; **Figure 1**) in mice that had performed the NSF task. Taken together, our results indicate that the increased neuronal activities in the aPVT and NAc can only be observed in mice that had performed the NSF task, implicating the aPVT and NAc are involved in food foraging but not novelty seeking in mice exploring a novel open field.

### Activation of aPVT Glutamatergic Projections to NAc Promotes NSF Behavior

**FIGURE 2:**
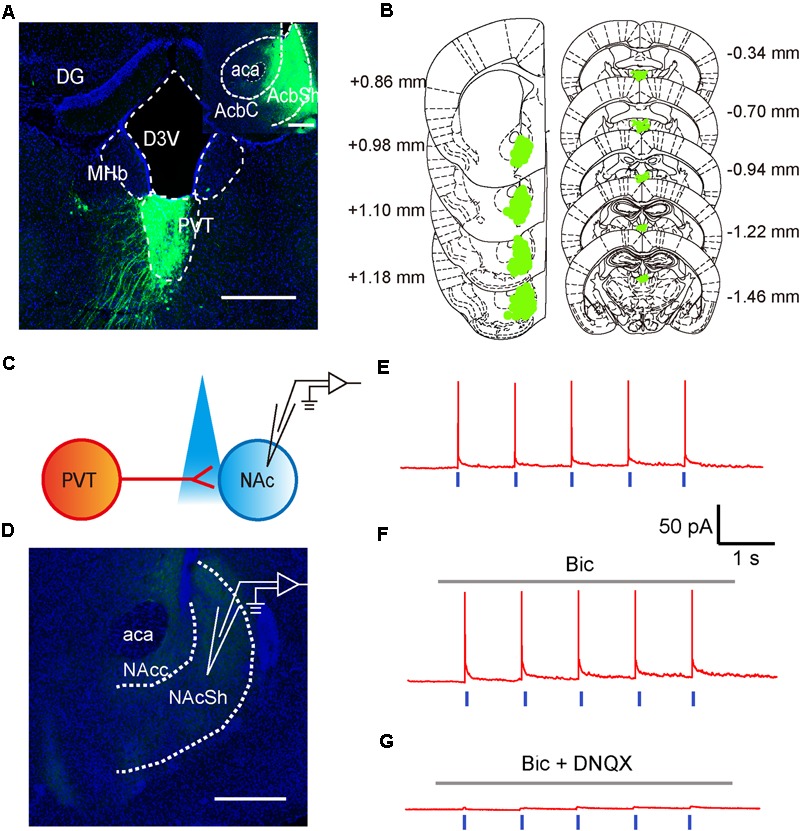
Glutamatergic neurons in the aPVT anatomically and functionally innervate the MSNs in the NAc. **(A)** Representative confocal image showing the retrograde labeling of retro-AAV2-CMV-GFP (injected in the NAc) in the aPVT. Inset, retro-AAV2-CMV-GFP virus injection site in the NAc. Scale bar = 500 μm. **(B)** Illustration of the virus infection sites in the NAc and retro-labeled regions in the aPVT overlayed on brain sections adapted from Paxinos and Franklin (2008). Numbers attached to the brain sections indicate the section position posterior to Bregma. **(C)** Schematic of electrophysiological recording paradigm. **(D)** Representative confocal image showing the recording site in the slices of mouse brain. Scale bar = 500 μm. **(E)** Representative trace of action potentials recorded from an MSN evoked by light stimulation at 1 Hz in the NAc. **(F,G)** Representative traces of action potentials evoked by light stimulation at 1 Hz in the NAc in the presence of bicuculine (bic) **(F)** or bic and DNQX **(G)**.

To test if neurons in the aPVT project to the NAc, we next investigated the anatomical and functional connections between neurons in the aPVT and NAc by retrograde tracing and optogenetic stimulation. Retro-rAAV2 is a newly developed AAV2 variant that enables robust retrograde access to projection neurons (Tervo et al., 2016). Hence, we injected the retro-AAV2-CMV-GFP virus in the NAc. The infection area was limited to an area from Bregma +1.18 to + 0.86 mm in the NAc. Strong retrograde viral signals were detected in the PVT area from Bregma -0.34 to -1.46 mm that includes the aPVT (Bregma -0.22 mm to -0.94 mm) (**Figures 2A,B**). We next investigated the functional connections between the aPVT glutamatergic neurons and NAc MSNs by injecting the AAV-CaMKIIα-ChR2-mCherry virus into the aPVT. Viral signals were easily detectable in the fibers terminated in the NAc. We then recorded from MSNs adjacent to the labeled terminals in the NAc (**Figures 2C,D**). As shown in **Figure 2E**, light-evoked action potentials (APs) were time-locked to the light stimuli. Administration of the GABA_A_ receptor antagonist BMI (10 μM) had no effect on light-evoked APs (**Figure 2F**); however, additionally applying the AMPA receptor antagonist DNQX (10 μM) abolished the light-evoked APs (**Figure 2G**). These results demonstrate there are glutamatergic projections from the aPVT neurons to the NAc MSNs.

**FIGURE 3:**
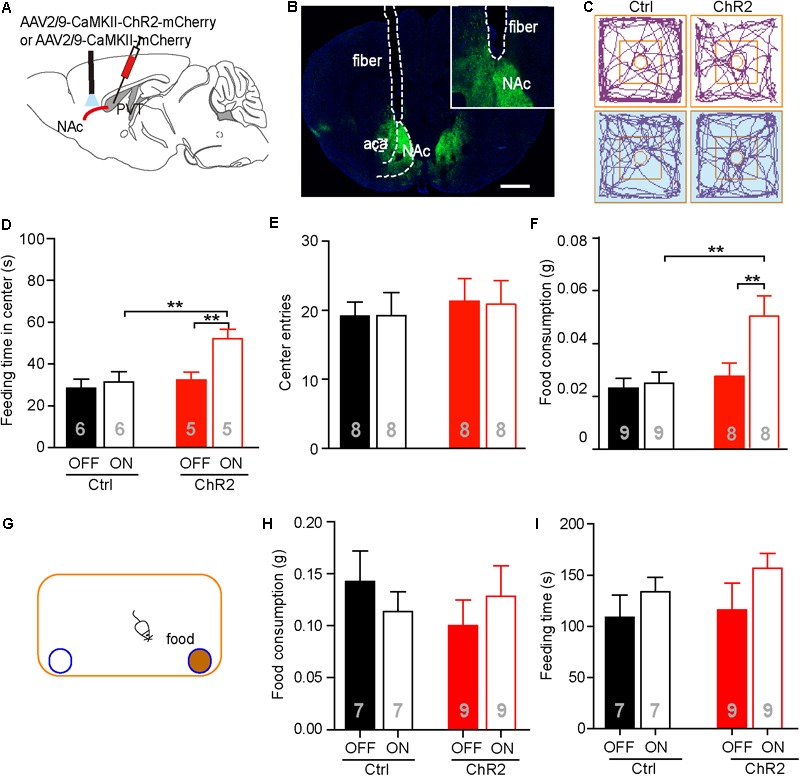
Activation of the aPVT glutamatergic projections to the NAc promotes NSF behavior. **(A)** Schematic diagram (upper panel) showing the positions of viral injection and laser stimulation. **(B)** Representative image (lower panel) showing the position of an optic fiber above the NAc. **(C)** Representative tracings of control and ChR2 mice performing the NFS task under the conditions of laser off (left) and laser on (right, blue shade). Small circles in the middle represent food placement; squares in the center show the central exploration area. **(D–F)** Quantification of the time spent in the central area **(D)**, the number of entries into the center **(E)**, and food consumption **(F)** for control and ChR2 mice under the conditions of laser off or laser on. **(G)** Schematic diagram showing the apparatus in which the normal feeding test is performed. Blue circle filled with brown represents a dish containing a food pellet. **(H,I)** Quantification of food consumption **(H)** and feeding time **(I)** for control and ChR2 mice under the conditions of laser off or laser on. ^∗∗^*p* < 0.01 (two-way ANOVA with *post hoc* Holm-Sidak test). Data are mean ± SEM.

It has been reported that the PVT-NAc circuit is involved in regulation of sucrose-seeking in an operant behavior (Labouebe et al., 2016). Based on the observation that the neuronal activities were increased in the aPVT and NAc immediately after an animal had performed the NSF task, we next investigated if activation of the aPVT-NAc circuit promoted food-seeking in a novel environment. We injected the AAV-CaMKIIα-ChR2-mCherry (ChR2 group) or AAV-CaMKIIα-mCherry (control group) virus into the aPVT, and then photostimulated viral-labeled axonal terminals in the NAc while a mouse was performing the NSF task (**Figures 3A,B**). Two-way ANOVA revealed a significant effect of laser (on vs. off) (*F*_(1,18)_ = 6.342, *p* = 0.021) and group (control vs. ChR2) (*F*_(1,18)_ = 6.848, *p* = 0.017) on the feeding time in center. *Post hoc* Holm-Sidak test further showed that photostimulation dramatically increased the feeding time in center when a mouse in the ChR2 group (*p* < 0.01, *n* = 5) but not in the control group (*p* > 0.05, *n* = 6) was performing the NSF task (**Figures 3C,D**). Mice in the ChR2 group spent more feeding time in center than those in the control group in response to laser stimulation (*p* < 0.01, *n* = 5 and 6 for ChR2 and control groups, respectively), whereas in baseline conditions (laser off), mice in the control and ChR2 groups spent same time in the center (*p* > 0.05; **Figures 3C,D**). However, there were no main effect of laser (on vs. off) (*F*_(1,28)_ = 0.160, *p* = 0.692) or group (ChR2 vs. control) (*F*_(1,28)_ = 0.0464, *p* = 0.831) and interaction effect between laser and group (*F*_(1,28)_ = 0.0464, *p* = 0.831) on center entries tested by two-way ANOVA (*n* = 8 and 8 for control and ChR2 groups, respectively; **Figures 3C,E**). These results implicate that activation of the aPVT-NAc circuit may promote feeding but not central exploration in a novel environment. Indeed, two-way ANOVA displayed a significant effect of laser (on vs. off) (*F*_(1,30)_ = 5.427, *p* = 0.027) and group (control vs. ChR2) (*F*_(1,30)_ = 7.648, *p* = 0.010) on food consumption in response to photostimulation during the 5 min of the NSF test. In baseline laser off conditions, mice in the control and ChR2 groups consumed the same amount of food as shown by *post hoc* Holm-Sidak test (*p* > 0.05, *n* = 8 and 9 for ChR2 and control groups, respectively; **Figure 3F**). In laser on conditions, mice in the ChR2 group consumed more food than those in the control group (*p* < 0.01, *n* = 8 and 9 for ChR2 and control groups, respectively; **Figure 3F**). Laser stimulation only significantly enhanced food consumption in ChR2 mice (*p* < 0.01, *n* = 8). These results indicate that activation of the aPVT-NAc circuit in mice increases food intake in a novel open field containing food in the center.

We next tested whether activation of the aPVT-NAc pathway promoted feeding in a familiar, less stressful environment. We recorded food consumption and feeding time of moderately fasted control or ChR2 mice within a 5-min period in a regular mouse cage, with or without activating the aPVT-NAc circuit (**Figure 3G**). Food consumption in the familiar environment was generally higher than that in the open field (**Figures 3F,H**). However, neither food consumption (two-way ANOVA; *F*_(1,28)_ = 0.273, *p* = 0.605, control vs. ChR2 group; *F*_(1,28)_ = 0.000, *p* = 0.986, laser off vs. laser on group; *F*_(1,28)_ = 1.178, *p* = 0.287, interaction; *n* = 7 and 9 for control and ChR2 groups, respectively; **Figure 3H**) nor feeding time (two-way ANOVA; *F*_(1,28)_ = 0.0.544, *p* = 0.467, control vs. ChR2 group; *F*_(1,28)_ = 0.2.542, *p* = 0.122, laser off vs. laser on group; *F*_(1,28)_ = 0.156, *p* = 0.696, interaction; *n* = 7 and 9 for control and ChR2 groups, respectively; **Figure 3I**) within this 5-min period was significantly altered by activation of the aPVT-NAc circuit. The results indicate that activation of the aPVT-NAc pathway exerts no influence on feeding behavior in a short period in a less stressful environment. Taken together, our results showed that activation of the aPVT-NAc circuit in mice increases food intake exclusively in a stressful environment.

### Activation of the aPVT-NAc Circuit Does Not Affect Novelty Seeking and Anxiety Level

**FIGURE 4:**
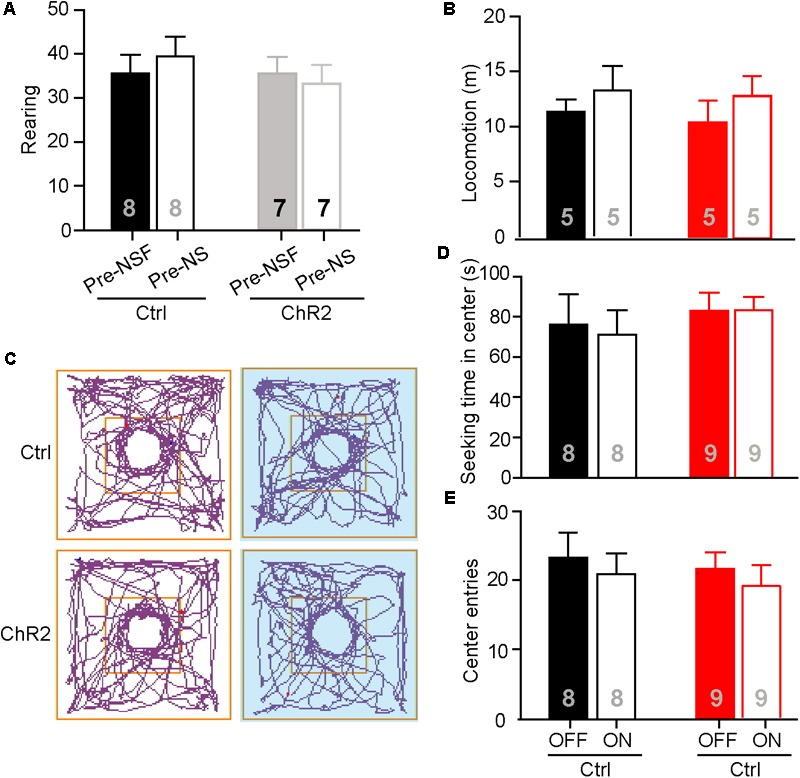
Activation of the aPVT-NAc circuit does not affect novelty seeking in mice. **(A)** Quantification of the number of rearing of control and ChR2 mice before (pre-NSF) and 7 days after (pre-NS) the NSF test. **(B)** Quantification of locomotive distance for control and ChR2 mice under the conditions of laser off and laser on. **(C)** Representative tracings of control and ChR2 mice performing the NS task under the conditions of laser off (left) and laser on (right, blue shade). Small circles in the middle represent novel object placement; squares in the center show the central exploration area. **(D,E)** Quantification of the time spent in the central area **(D)** and the number of entries into the center **(E)** for control and ChR2 mice under the conditions of laser off and laser on. Data are mean ± SEM.

The neuronal activities examined by c-Fos staining in the aPVT and NAc remained unaltered in mice that had performed the NS or OF task. To further test if the aPVT-NAc circuit is involved in OF exploration and NS, we photostimulated the aPVT-NAc circuit when an animal was performing the OF or NS task 7 days after the initial NSF task. It is important to determine whether the open field apparatus is still novel to the mice that had experienced the device in the prior NSF test. To this end, we video-recorded two open field sections for each mouse and analyzed the rearing behaviors before (pre-NSF) and 7 days after (pre-NS) the initial NSF test. No significant difference in the number of rearing times between the pre- and post-NSF tests was found in control and ChR2 groups (two-way ANOVA; *F*_(1,24)_ = 0.639, *p* = 0.432, control vs. ChR2 group; *F*_(1,24)_ = 0.0489, *p* = 0.827, pre-NSF vs. post-NSF group; *F*_(1,24)_ = 0.486, *p* = 0.0492, interaction; *n* = 8 and 7 for control and ChR2 groups, respectively; **Figure 4A**). As rearing behavior is believed to be linked to the cognitive functions (Lever et al., 2006), these data suggest that mice taken a prior NSF test will not get used to the open field apparatus 7 days later. Besides, we found that there were no main effect of laser (on vs. off) (*F*_(1,16)_ = 1.425, *p* = 0.250) or group (ChR2 vs. control) (*F*_(1,16)_ = 0.173, *p* = 0.683) and interaction effect between laser and group (*F*_(1,16)_ = 0.0532, *p* = 0.821) on the locomotor activities during the OF sections (*n* = 5 for each group; **Figure 4B**). We then tested whether photostimulating (laser on vs. off) the aPVT-NAc circuit altered the NS behavior in mice (control vs. ChR2) when a neutral novel objective was placed in the center of the open field. We found that neither the exploration time in the center (two-way ANOVA; *F*_(1,30)_ = 0.625, *p* = 0.435, control vs. ChR2 group; *F*_(1,30)_ = 0.0221, *p* = 0.883, laser off vs. laser on group; *F*_(1,30)_ = 0.0919, *p* = 0.764, interaction; *n* = 8 and 9 for control and ChR2 groups, respectively; **Figures 4C,D**) nor the number of entries into the center (two-way ANOVA; *F*_(1,30)_ = 0.289, *p* = 0.595, control vs. ChR2 group; *F*_(1,30)_ = 0.000133, *p* = 0.991, laser off vs. laser on group; *F*_(1,30)_ = 0.640, *p* = 0.430, interaction; *n* = 8 and 9 for control and ChR2 groups, respectively; **Figures 4C,E**) was changed by activation of the aPVT-NAc circuit. Together, the results clarify that the increased time spent in the center of the open field presented with a food pellet caused by activation of the aPVT-NAc circuit in the NSF test is due to a promotion of the feeding behavior but not an enhancement of the exploration behavior in a novel open field.

**FIGURE 5:**
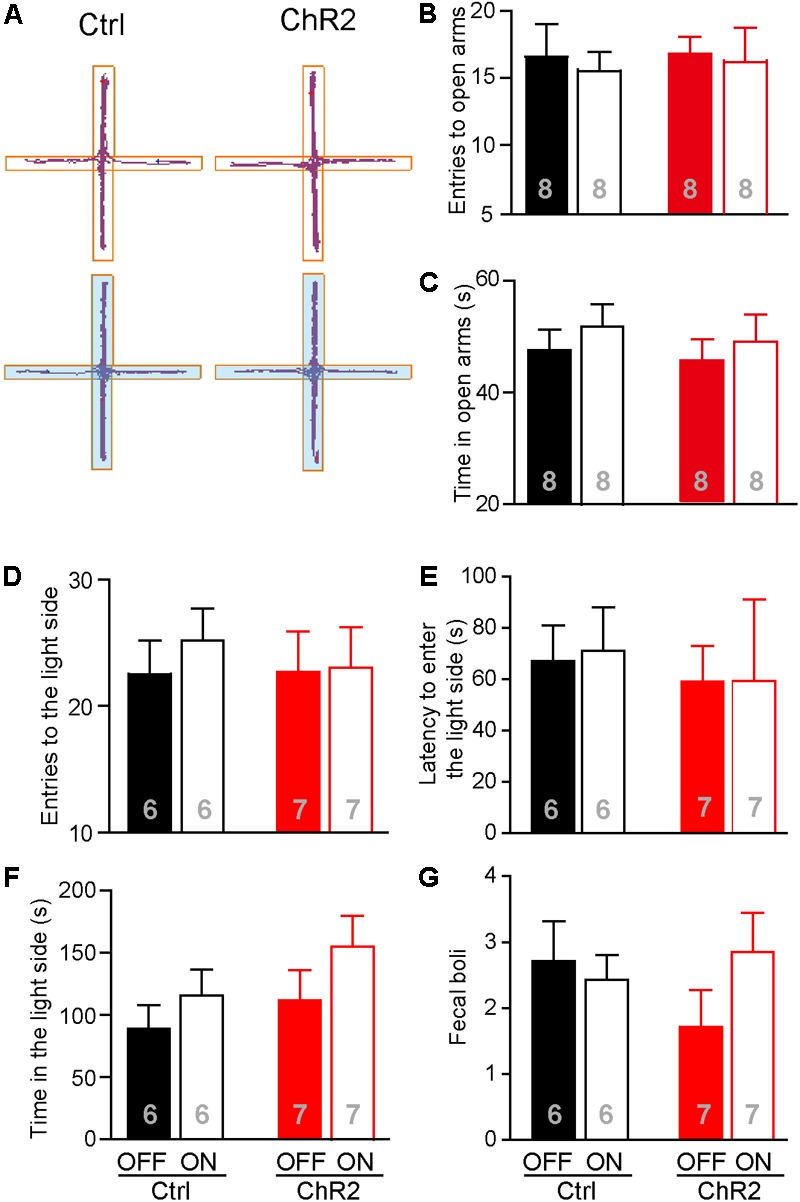
Activation of the aPVT-NAc circuit does not affect the anxiety level in mice. **(A)** Representative tracings of control and ChR2 mice performing the EPM test under the conditions of laser off (upper) or laser on (lower, blue shade). Open arms are horizontal in the panel. **(B,C)** Quantification of the number of entries into the open arms **(B)** and the time spent in the open arms **(C)** for control and ChR2 mice under the conditions of laser off or laser on. **(D–G)** Analysis of the number of entries into the light compartment **(D)**, the latency of entering into the light compartment **(E)**, the time spent in the light compartment **(F)**, and the number of fecal boli produced **(G)** in the light–dark box test for control and ChR2 mice under the conditions of laser off or laser on. Data are mean ± SEM.

Increased food intake associated with activation of the aPVT-NAc projection in mice approaching food in a novel open field may also be due to a reduced anxiety level in these animals. We thus investigated whether activation of the aPVT-NAc circuit (laser on vs. off) affects the anxiety level in control and ChR2 mice. We performed the EPM test and light–dark box test in control and ChR2 mice to evaluate their anxiety levels (Mombereau et al., 2004; Griebel and Holmes, 2013; Haller et al., 2013). In the EPM test, we found that photostimulating the aPVT-NAc circuit did not change the number of entries to the open arms in both control and ChR2 mice (two-way ANOVA; *F*_(1,28)_ = 0.0464, *p* = 0.831, control vs. ChR2 group; *F*_(1,28)_ = 0.160, *p* = 0.692, laser off vs. laser on group; *F*_(1,28)_ = 0.0464, *p* = 0.8312, interaction; *n* = 8 for each group; **Figures 5A,B**). In addition, there were no changes in the time spent in the open arms for either control or ChR2 mice no matter whether photostimulation is on or off (two-way ANOVA; *F*_(1,28)_ = 0.208, *p* = 0.652, control vs. ChR2 group; *F*_(1,28)_ = 0.304, *p* = 0.586, laser off vs. laser on group; *F*_(1,28)_ = 0.0147, *p* = 0.904, interaction; *n* = 8 for each group; **Figures 5A,C**). In the light–dark box test, photostimulation of the aPVT-NAc circuit did not change the number of entries into the light compartment (interaction of laser and group, *F*_(1,22)_ = 0.157, *p* = 0.695; main effect of laser, *F*_(1,22)_ = 0.242, *p* = 0.628; main effect of group, *F*_(1,22)_ = 0.106, *p* = 0.748, **Figure 5D**), the latency of entering into the light compartment (interaction of laser and group, *F*_(1,22)_ = 0.008, *p* = 0.925; main effect of laser, *F*_(1,22)_ = 0.007, *p* = 0.933; main effect of group, *F*_(1,22)_ = 0.204, *p* = 0.656, **Figure 5E**), the time spent in the light compartment (interaction of laser and group, *F*_(1,22)_ = 0.168, *p* = 0.686; main effect of laser, *F*_(1,22)_ = 2.313, *p* = 0.143; main effect of group, *F*_(1,22)_ = 1.9434, *p* = 0.177, **Figure 5F**), and the number of fecal boli produced (interaction of laser and group, *F*_(1,22)_ = 0.198, *p* = 0.173; main effect of laser, *F*_(1,22)_ = 0.303, *p* = 0.587; main effect of group, *F*_(1,22)_ = 0.260, *p* = 0.615, **Figure 5G**). However, there was a tendency that activation of the aPVT-NAc pathway increased the exploratory time in the light compartment as shown in **Figure 5F**, suggesting that activation of the aPVT-NAc pathway may facilitate habitation in a novel environment. Together, these results indicate that activation of the aPVT-NAc circuit does not generate significant alterations in the anxiety-like behavior in mice.

## Discussion

The main findings of the present study are: (1) the NSF test induces an increased number of c-Fos positive cells in the NAc and aPVT in mice, and stimulation of the aPVT-NAc circuit promotes the feeding behavior in a novel open environment; (2) the aPVT-NAc circuit is not recruited either in the novelty seeking or anxiety-related behavior. These data are the first to reveal a function of the aPVT-NAc circuit in regulating motivational feeding under approach-avoidance conflict.

Although stimulating the aPVT-NAc projection positively regulates motivated feeding behavior as shown in our study, activation of the PVT-NAc pathway has been linked to behavioral aversion in opiate withdrawn animals (Zhu et al., 2016). We believe this discrepancy may be due to a heterogeneous population of neurons in the PVT projecting to the NAc. Neurons in the PVT may function differently based on their locations and projections. For instance, activation of the orexin and substance P receptor signaling pathways in the aPVT increases alcohol intake in rodents (Barson et al., 2015, 2017). Activation of the aPVT-NAc pathway reduces sucrose-seeking in an operant task (Do-Monte et al., 2017). The PVP functions to inhibit hypothalamic-pituitary-adrenal activity under conditions of chronic stress (Bhatnagar et al., 2002). Generally speaking, the PVP mainly regulates stress-related behaviors (Chastrette et al., 1991; Bubser and Deutch, 1999; Heydendael et al., 2011), whereas the aPVT is involved in drug and natural reward seeking (Choi et al., 2010). Indeed, behavioral aversion observed by Zhu et al. (2016) is associated with the medial and posterior divisions of the PVT. Furthermore, different PVT projection neurons may target different MSNs in the NAc to trigger aversive or appetitive behavior (Millan et al., 2017). The aversive motivational consequences of drug withdrawal may primary be associated with chronic drug exposure-induced strengthening of the PVT inputs selectively targeting dopamine (DA) D2 receptor-expressing MSNs in the NAc (Zhu et al., 2016). On the other hand, appetitive motivational behavior may be linked to the PVT projections selectively onto DA D1 receptor-expressing MSNs in the NAc (Millan et al., 2017). Further location- and cell-type-specific manipulations of the PVT-NAc pathway are required to precisely elucidate the detailed mechanism underlying motivated feeding behavior in future studies.

Motivational feeding in a novel environment may be initiated by food with high reward value and/or reduced inhibitory control of feeding. The level of DA in the NAc after food consumption may represent a measure for food value and provide a rough proxy for the amount of motivation an animal has to ignore risks and costs and forage food (Cassidy and Tong, 2017). Reduced DA level in the NAc is associated with reduced drive to obtain cocaine and sucrose (Wellman et al., 2007; Davis et al., 2008), while increased DA level in the NAc promotes food foraging (Meye and Adan, 2014; Sackett et al., 2017). The PVT glutamatergic projections within the NAc shell are apposed to the DA fibers (Pinto et al., 2003) and glutamate released from these PVT terminals acts on ionotropic glutamate receptors in the DA fibers to induce DA efflux (Parsons et al., 2007). Thus, we hypothesize that activation of the aPVT-NAc projection may increase the DA level in the NAc and thereby raise the reward value of food. It will be interesting to establish the relationship between the food reward value and the aPVT-NAc activation using the sucrose preference test in future studies.

Activation of the aPVT-NAc pathway may also suppress the inhibitory control of feeding to promote food foraging in a novel environment. Innate fear for the large, open field and a high anxiety level may prevent mice from foraging food in the open field apparatus. Serotonin is implicated in mood regulation, especially anxiety and depression. Mice lacking 5-HT_1A_ receptors exhibit increased anxiety level (Parks et al., 1998; Gross et al., 2000), reduced locomotor activity, and abnormal emotional behaviors (Ansorge et al., 2004). 5-HT_1A_ receptors are abundant in the midline thalamic nuclei in humans (Varnas et al., 2004). Majority of calbindin-expressing neurons are also double-labeled for 5-HT_1A_ receptor in the PVT in rats (Oliver et al., 2000; Aznar et al., 2003). These studies raise the possibility that serotonergic inputs to the PVT may be involved in regulating anxiety (Hsu et al., 2014). Reduced serotonergic function in the PVT thus may represent a negative control mechanism for motivational feeding. Increasing the activity of PVT neurons may increase serotonergic function in the PVT, hence suppressing the negative motivation for food foraging. Although unlikely based on our EPM results, it is still worth testing the plausible positive relationship between serotonergic function in the PVT and motivational feeding in future studies.

Another interesting finding of the current study is that the feeding behavior induced by photostimulating the aPVT-NAc pathway is not precisely locked to photostimulation. This is different from instant consummatory feeding behavior evoked by stimulating the GABAergic fibers from zona incerta in the PVT (Zhang and van den Pol, 2017) or from the lateral hypothalamus (LH) in the paraventricular hypothalamus (Wu et al., 2015). Therefore, the aPVT-NAc connection may not function as a feeding circuitry that directly regulates the consummatory behavior. Instead, this circuit may facilitate appetitive feeding behavior via dynamically regulating the motivation to eat in a novel environment. The LH has long been identified as a critical structure for feeding and motivational behaviors (Hoebel and Teitelbaum, 1962; Margules and Olds, 1962; Jennings et al., 2013). Diverse types of neurons within the LH have been proposed to function differently because of their distinct anatomical and molecular connections to downstream targets (Stuber and Wise, 2016). The orexin/hypocretin fibers originated from the LH can be readily identified in the PVT and are involved in drug seeking and motivated feeding (Kirouac et al., 2005). Cocaine- and amphetamine-regulated transcript (CART) neurons in the LH projecting to the PVT may control food-seeking behavior under natural conditions (Choudhary et al., 2017). An infusion of CART into the PVT attenuates cocaine-primed reinstatement (James et al., 2010). The PVT neurons then send the glutamatergic fibers to the NAcSh, the canonical site for reward, presynaptically regulating DA release in the NAcSh (Parsons et al., 2007). Based on these observations, we believe there is a great chance the LH-PVT-NAc circuit is involved in appetitive feeding behavior in a novel environment.

In addition to glutamatergic neurons in the PVT, various types of neurons in the ventral tegmental area (VTA) and medial prefrontal cortex (mPFC) also project to the NAc. The VTA is widely regarded as an integral part of the drug and natural reward circuitry of the brain (Narayanan et al., 2010; Morales and Margolis, 2017). Consumption of a palatable diet in a short period has been shown to rapidly enhance excitatory inputs onto DA neurons in the VTA, hence facilitating future food approach behavior of mice in a risky environment (Liu et al., 2016). Optogenetic induction of phasic firing in NAc-projecting VTA DA neurons, which may encode a reward prediction error, increases social avoidance and reduces sucrose preference in mice (Chaudhury et al., 2013). DA neurons in the VTA also react to aversive stimuli mostly by transient silencing, as inhibition of these neurons projecting to D2 receptor-expressing MSNs in the NAc induces aversive behavior in mice (Danjo et al., 2014). Therefore, there is a great chance that the VTA-NAc circuit is actively involved in evaluating food reward in a risky environment. The mPFC is well-known to function as a center for risk-based decision-making (Bechara, 2005; Lopez-Ramos et al., 2015). It has been reported that the mPFC-NAc circuit encodes the decision to initiate or suppress reward seeking when a risk is encountered (Kim et al., 2017), suggesting mPFC may play an important role in food foraging in a novel environment. Future studies are needed to test the involvement of these important circuit in regulating consummatory feeding behavior under approach-avoidance conflict.

## Conclusion

The current work depicts a pivotal function of the PVT-NAc circuit in motivated feeding behavior under approach-avoidance conflict. This function is essential for survival in the wild where food is scarce. Overactivation or inhibition of this pathway may lead to compulsive eating or anorexia, respectively.

## Author Contributions

JC and JW performed behavioral tests and immunostaining. XM performed the electrophysiology recordings. JC and RU analyzed the data. Y-DZ and JC designed the research. JC, YS, and Y-DZ wrote the paper. All authors contributed to manuscript revisions.

## Conflict of Interest Statement

The authors declare that the research was conducted in the absence of any commercial or financial relationships that could be construed as a potential conflict of interest.
